# Nutritional status and growth of indigenous Xavante children, Central Brazil

**DOI:** 10.1186/1475-2891-11-3

**Published:** 2012-01-11

**Authors:** Aline A Ferreira, James R Welch, Ricardo V Santos, Silvia A Gugelmin, Carlos EA Coimbra

**Affiliations:** 1 Escola Nacional de Saúde Pública, Fundação Oswaldo Cruz, Rio de Janeiro, Brazil; 2 Departamento de Nutrição Social, Universidade do Estado do Rio de Janeiro, Rio de Janeiro, Brazil; 3 Departamento de Antropologia, Museu Nacional, Universidade Federal do Rio de Janeiro, Rio de Janeiro, Brazil

## Abstract

**Background:**

The aim of this study was to characterize the nutritional status of Xavante Indian children less than 10 years of age in Central Brazil and to evaluate the hypothesis of an association between child nutrition and socioeconomic differentiation in this population.

**Methods:**

A cross-sectional study was conducted in July 2006 that included all children under the age of 10 from the Xavante village Pimentel Barbosa in Mato Grosso, Brazil. The data collected included weight, height, and sociodemographic information. Sociodemographic data were used to generate two indices ("income" and "wealth") and to determine the proportion of adults in each household. Descriptive analyses were performed for weight-for-age (W/A), height-for-age (H/A), and weight-for-height (W/H) using the NCHS and the WHO growth references. Univariate and multivariate analyses were conducted using H/A and W/A as a response variables.

**Results:**

Of a total of 246 children under the age of ten residing in the village, 232 (94.3%) were evaluated. Following the NCHS reference, 5.6% of children under the age of ten presented low W/A and 14.7% presented low H/A. Among children under the age of five, deficit percentages for weight and height were 4.5% and 29.9%, respectively, following the WHO curves. Among children < 2 years of age, H/A index variability was found to be directly related to child's age and inversely related to the proportion of adults in the household. Maternal BMI was positively associated with growth for children from 2 to 4 years of age, explaining 11.5% of the z-score variability for the H/A index. For children 5 years of age and older, the wealth index and maternal height were positively associated with H/A. No significant associations were found using W/A as the dependent variable.

**Conclusion:**

This study demonstrated that undernutrition, in particular linear growth deficit, is a notable health issue for Xavante children. These findings contrast with the nutritional profile observed among Brazilian children nationally, which is characterized by a sharp decline in child undernutrition in recent decades, even in the poorest regions of the country. This discrepancy calls attention to the persistent health disparities that exist between indigenous and non-indigenous people in Brazil.

## Background

Rapid declines in child undernutrition have been recorded since the 1980s in regions of Brazil historically characterized by the worst health indicators, such as the north and northeast. Such reductions have been interpreted mainly as the result of the universalization of access to health services and improved maternal education and sanitation [1-3].

A different scenario exists among the indigenous peoples in Brazil. Studies conducted in the last two decades among different ethnic groups in the Brazilian Amazon and Central Brazil have demonstrated high prevalences of chronic undernutrition in children, often affecting more than a quarter of those below five years of age [4-6]. The results of the First National Survey of Indigenous People's Health and Nutrition, which evaluated the Brazilian indigenous population in 2008-2009, confirmed the scenario previously outlined by local case studies, pointing to height-for-age deficit as a highly prevalent nutritional disorder among indigenous children [7]. However, little is known about the determinants of child nutritional status in indigenous societies.

Available epidemiological studies of indigenous children in Brazil point to the predominance of infectious diseases, with diarrhea and acute respiratory infections being the principal causes of illness and death among children under five years of age [8-10]. Infant mortality rates are high, often above 50 per thousand [11-13], and the coverage of relevant child health programs, including prenatal care and vaccination, is irregular [7].

The recent history of the Xavante people has been marked by rapid socioeconomic and environmental change, with repercussions for their food systems and nutritional status. Studies conducted in different Xavante communities since the 1990s have drawn attention to the importance of nutritional disorders, including weight and height deficits as well as anemia in the epidemiological profile of children [14-17]. Little is known about the determinants of these conditions. In an investigation of the nutritional status of adult residents of Pimentel Barbosa village, the largest village in the Pimentel Barbosa Indigenous Reserve, Welch et al. [18] identified an accelerated process of nutritional transition with high prevalences of overweight and obesity in adults of both sexes. This study also documented associations between anthropometric indicators of overweight or obesity with internal socioeconomic differentiation within the community, a pattern that did not exist before. It is worth noting that the Xavante population from the Pimentel Barbosa Indigenous Reserve has high infant mortality rates (average of 83.3/1000 between 1999 and 2004) and lives under precarious sanitary conditions [19].

The objective of the present study was to describe the nutritional status and analyze factors associated with growth of Xavante children less than ten years of age residing at Pimentel Barbosa village. We evaluated the hypothesis that factors associated with socioeconomic differentiation and demographic characteristics are associated with child nutrition, as has been suggested elsewhere for adults in the same community [18].

### Population and Method

This cross-sectional study was conducted among the Xavante population of Pimentel Barbosa village, also known at the time of the survey as Etênhiritipá, located in the Pimentel Barbosa Indigenous Reserve, Mato Grosso State. Fieldwork was conducted in July 2006 as part of a study that focused on the epidemiology of tuberculosis [20]. At the time of fieldwork, the population totaled 565 individuals, of which 246 (43.5%) were less than ten years of age and 139 (24.6%) were less than five years of age. The study sought to include all children less than ten years of age residing in the village. For this reason, no specific sampling techniques were employed.

#### Demographic and anthropometric data

A population census was conducted by means of household interviews. Children's birthdates were obtained from the local health service, which maintained birthdate lists for all village residents. For improved accuracy, the birthdates of mothers of evaluated children were cross-referenced with the demographic records of anthropologist Nancy M. Flowers, who worked in the community from the 1970s to 1990s. Weight and height measures of participants were collected at a temporary research clinic assembled in the village school by a single observer (AAF) following Lohman's recommendations [21]. Children under 24 months of age were weighed and measured using SECA (Hamburg, Germany) scales (model 745) and anthropometers (model 475) , with 0.1 kg and 0.1 cm precision, respectively. Children two years of age and older were evaluated using SECA digital scales (model 770) and GPM (Zurich, Switzerland) portable anthropometers, with 0.1 kg and 0.1 cm precision, respectively. Maternal data (height, weight, and body mass index) were obtained using similar methodology and instruments, as described by Welch et al. [18].

#### Socioeconomic and demographic indicators

In order to characterize socioeconomic differentiation within the study population, data regarding monthly household income ("income") and the possession of durable industrial goods ("wealth") were collected following the methodology previously described by Welch et al. [18]. These methods are summarized below.

Structured household interviews were conducted by one of the authors (JRW) in order to obtain information regarding household income and wealth. In the case of income, information was solicited about regular sources (wages, pensions, retirement benefits, and others) for all members of the household. The values of reported income sources were retrospectively estimated through independent interviews. In the case of wealth, durable industrial goods owned by the members of each household were identified using a comprehensive list of goods (motorcycle, satellite dish, television, VCR player, DVD player, tape recorder, portable radio, cell phone, standard camera, digital camera, gas stove, sewing machine, firearm, fishing net and bicycle) that was compiled during a preliminary survey. The market values of these items were estimated through interviews with owners as well as a survey of local businesses.

Total household income was calculated as the sum of the values of all sources of income reported for the members of each household. Similarly, total household wealth was calculated as the sum of the values of all industrial goods reported for each household. Income and wealth indices for socioeconomic status were calculated by dividing the total value for each household by the highest value obtained in the village, such that values varied between 0 and 1. These values were attributed to all members of each household.

An index was also calculated to characterize households in terms of relative number of adult residents. The proportion of adults in each household was obtained by dividing the number of adults between 18 and 50 years of age by the total number of residents in the same household.

The concept of "household" employed in this study was based on the domestic unit that prepares food together. At the time of data collection, Pimentel Barbosa village consisted of 34 houses distributed in a semicircle. The Xavante typically reside in extended family groups, which favors the sharing of financial and dietary resources. The number of members in each house often reaches 20 to 30 people or more. A specific location for food preparation is typically associated with each house. These kitchens range from open fires on the earth floor inside a house to, more commonly, external structures with or without walls and covered with palm thatch, located in the immediate vicinity of a house. In some cases, the members of an extended family may live in two adjacent houses and share a kitchen. For the purpose of this study, a household was considered to be an extended family group residing in one or two houses and sharing a single kitchen. The total number of households was 30 and all households had at least one child < 10 years old.

#### Data analysis

Height, weight, and age data were used to calculate z-scores for the height-for-age (H/A), weight-for-age (W/A), and weight-for-height (W/H) indices according to reference curves provided by the National Center for Health Statistics (NCHS) [22,23] and the World Health Organization (WHO) [24]. Z-scores were calculated with the Anthro program (WHO Anthro, Geneva, Switzerland). Cutoff points followed those proposed by the WHO for the diagnosis of low H/A (z-scores < -2.00), low and high W/A (z-scores < -2.00 and > 2.00, respectively), and low and high W/H (z-scores < - 2.00 and > 2.00, respectively) [24]. Data were analyzed using the two growth references in order to produce prevalences comparable to those reported in previous studies of indigenous children in Brazil, which used one or both of the references.

Concerning univariate statistical analyses, the chi-square test was used to evaluate proportions. For multivariate analyses, the dependent variables (H/A and W/A) were classified following the NCHS reference population for children < 10 years of age, according to three age ranges: < 2 years, ≥ 2 and < 5 years, and ≥ 5 and < 10 years. Independent variables included age, sex, socioeconomic status, proportion of adults in the household, and maternal data. The socioeconomic status indices income and wealth were used in independent models. Sex, income, and wealth were expressed as categorical variables, with the last two subdivided into higher and lower strata according to the median.

The following procedures were performed in independent models using the socioeconomic indices income and wealth. Following verification of parametric premises, the contribution of each independent variable to H/A and W/A was assessed separately using multivariate linear regression. Variables that presented p-values < 0.30 were selected for the next step. In the multivariate analysis, the final model was arrived at through stepwise regression, employing manual followed by automatic backward elimination. The degree of linear correlation between independent variables and the response variable was analyzed with Pearson's correlation coefficient. In order to verify the final model, collinearity between independent variables was assessed using the variance inflation factor (VIF) with a cutoff point of VIF ≤ 10. The contribution of variables was assessed using ANOVA, F-test, and analysis of residuals. All results were considered significant at p < 0.05.

All data analyses other than the calculation of z-scores were performed using the software programs Statistical Package for the Social Sciences (SPSS) for Windows, version 16.0 (SPSS Inc., Chicago, IL, USA) and R, version 2.4.1 (http://www.r-project.org).

#### Permissions and ethics

The study was approved by the Research Ethics Committee of the National School of Public Health, Oswaldo Cruz Foundation (Fundação Oswaldo Cruz), and by the Brazilian National Committee for Ethics in Research (Comissão Nacional de Ética em Pesquisa). Research was authorized by the National Indian Foundation (Fundação Nacional do Índio). The project was presented to and approved by community leaders at a public meeting in the village. Furthermore, parents were present during the collection of anthropometric data and were allowed to choose not to participate on behalf of their children.

## Results

Of a total of 246 children under ten years of age, 232 (94.3%) were evaluated. Most instances of non-participation were due to absence from the village at the time of evaluation (5.3% of the study population). Only one child (0.4%) was not included due to the decision by her parents to not participate in the study. Height was not determined for six children (2.5%) due to their agitation during measurement. Additionally, the process of data cleansing resulted in the exclusion of other children for specific variables. As a result, the final database permitted analysis of 223 children (90.6%) for the W/H index, 232 (94.3%) for W/A, and 225 (91.5%) for H/A.

According to the NCHS reference curves, 5.6% of children < 10 years of age exhibited undernutrition based on W/A z-scores < -2.00 and 14.7% based on H/A z-scores < -2.00. Additionally, 40.4% were considered at nutritional risk based on H/A z-scores ≥ -2.00 and < -1.00. H/A frequencies were significantly different according to sex, with girls presenting slightly higher frequencies of z-scores in the ranges < -2.00 and -2.00 ≤ z-scores < -1.00 (p = 0.010). Girls had slightly higher frequencies of low W/A (z-scores < -2.00) than boys; however, this difference was not statistically significant (p = 0.310). There were no cases of low weight-for-height, while excess weight relative to height (W/H z-scores > 2.00) was observed in 1.8% of male and female children (Table 1).

**Table 1:** Distribution (absolute and relative) of z-scores for the indices height-for-age (H/A), weight-for-age (W/A), and weight-for-height (W/H) of Xavante children < 10 years of age, according to sex, following NCHS reference.

Z-scores	H/A			W/A			W/H		
	
	Sex			Sex			Sex		
						
	M	F	Total	M	F	Total	M	F	Total
	n (%)	n (%)	n (%)	n (%)	n (%)	n (%)	n (%)	n (%)	n (%)
**< -2.00**	16* (14.2)	17* (15.2)	33 (14.7)	5 (4.3)	8 (6.9)	13 (5.6)	0 (0.0)	0 (0.0)	0 (0.0)
**-2.00 ≤ z < -1.00**	45* (39.8)	46* (41.1)	91 (40.4)	32 (27.6)	32 (27.6)	64 (27.6)	6 (5.3)	4 (3.6)	10 (4.5)
**-1.00 ≤ z ≤ 2.00**	52* (46.0)	49* (43.7)	101 (44.9)	79* (68.1)	76* (65.5)	155 (66.8)	104 (92.9)	105 (94.6)	209 (93.7)
**> 2.00**	0 (0.0)	0 (0.0)	0 (0.0)	0 (0.0)	0 (0.0)	0 (0.0)	2 (1.8)	2 (1.8)	4 (1.8)
**Total**	113	112	225	116	116	232	112	111	223

Pimentel Barbosa village, Brazil, 2006.

Obs.: (*) significant results, 1 d.f. (p < 0.05).

The relative distribution of undernutrition (z-scores < -2.00 for W/A and H/A) was analyzed according to the NCHS references in children under ten years of age, by age group in months (Table 2). The age range ≥ 36 and < 48 months showed the highest percentage of height deficit (35.0%), which was followed by the group ≥ 6 and < 12 months of age (30.8%). Weight deficit prevalences were higher among children in the age groups ≥ 6 and < 12 months (15.4%) and ≥ 12 and < 24 months (20.0%). Weight deficit was not observed in children under 6 months and children ≥ 5 years old. No significant differences in undernutrition prevalences were observed in any age range for W/A and H/A.

**Table 2:** Distribution (absolute and relative) of height and weight deficit values for the indices height-for-age (H/A) and weight-for-age (W/A) of Xavante children < 10 years of age, according to age, following NCHS reference.

Age in months	H/A		W/A	
	
	n	Low H/A*	n	Low W/A*
		**%**		**%**
				
**< 6.0**	16	0.0	16	0.0
**≥ 6.0 and < 12.0**	13	30.8	13	15.4
**≥ 12.0 and < 24.0**	24	20.8	25	20.0
**≥ 24.0 and < 36.0**	24	12.5	28	7.1
**≥ 36.0 and < 48.0**	20	35.0	20	5.0
**≥ 48.0 and < 60.0**	30	16.7	32	9.4
**≥ 60.0 and < 72.0**	15	6.7	15	0.0
**≥ 72.0 and < 84.0**	21	19.0	21	0.0
**≥ 84.0 and < 96.0**	15	6.7	15	0.0
**≥ 96.0 and < 108.0**	25	4.0	25	0.0
**≥ 108.0 and < 120.0**	22	9.1	22	0.0

**Total**	225	14.7	232	5.6

Pimentel Barbosa village, Brazil, 2006.

Obs.: (*) z-scores < -2.00.

Table 3 summarizes the nutritional profile of children less than 5 years of age according to the NCHS and WHO references. There were major differences in the prevalences according to the growth references. For children of both sexes, the use of the WHO reference resulted in a marked increase in the prevalence of low H/A (< -2.00) compared to the NCHS (from 18.9% to 29.9%). On the other hand, there was a decrease in the prevalence of low W/A (from 9.7% to 4.5%). For W/H, the frequency of z-scores < -2 was 0.0% for both references, while the prevalence of z-scores > 2 was 1.5% for both references (data not shown in the table). In general, these patterns were consistent for males and females.

**Table 3:** Distribution (absolute and relative) of z-scores for the indices height-for-age (H/A) and weight-for-age (W/A) of Xavante children < 5 years of age, according to sex and growth references.

z-scores	H/A						W/A					
	
	NCHS		Total	WHO		Total	NCHS		Total	WHO		Total
								
	Males	Females	n (%)	Males	Females	n (%)	Males	Females	n (%)	Males	Females	n (%)
	n (%)	n (%)		n (%)	n (%)		n (%)	n (%)		n (%)	n (%)	
**< -2.00**	11 (17.5)	13 (20.3)	24 (18.9)	23 (36.6)	15 (23.4)	38 (29.9)	5 (7.6)	8 (11.8)	13 (9.7)	2 (3.0)	4 (5.9)	6 (4.5)
**-2.00 ≤ z < -1.00**	30 (47.6)	31 (48.4)	61 (48.0)	20 (31.7)	34 (53.2)	54 (42.5)	23 (34.8)	20 (29.4)	43 (32.1)	20 (30.3)	18 (26.5)	38 (28.3)
**-1.00 ≤ z ≤ 2.00**	22 (34.9)	20 (31.3)	42 (33.1)	20 (31.7)	15 (23.4)	35 (27.6)	38 (57.6)	40 (58.8)	78 (58.2)	44 (66.7)	46 (67.6)	90 (67.2)
**> 2.00**	0 (0.0)	0 (0.0)	0 (0.0)	0 (0.0)	0 (0.0)	0 (0.0)	0 (0.0)	0 (0.0)	0 (0.0)	0 (0.0)	0 (0.0)	0 (0.0)
**Total**	63	64	127	63	64	127	66	68	134	66	68	134

Pimentel Barbosa village, Brazil, 2006.

Of the 225 children for whom H/A indices were calculated, 173 (76.9%) also had sufficient data for inclusion in linear regression analysis. Tables 4 and 5 present the variables showing significant associations with children's linear growth (H/A) according to age group. In children less than two years old, age (in months) and the proportion of adults in the household explained 34.5% of the variation in H/A in both the income and wealth models. There was an inverse relationship between linear growth and age in this age range. In other words, the younger the age of the child, the higher was his or her H/A. Within the same age range, it was observed that the higher the proportion of adults in the household, the higher the H/A of the child.

**Table 4:** Multivariate analysis of independent variables in relation to the response variable (height-for-age), using the socioeconomic index wealth, of Xavante children < 10 years of age, according to age, Pimentel Barbosa village, Brazil, 2006.

Age in years	Independent variables	Linear coefficient	Angular coefficient	p-value	Adjusted R^2^	F-test	p-value (F-test)
	Age		-0.078	0.000			
**< 2**		-1.770			0.345	12.067	0.000
	Proportion of adults		4.845	0.030			

**≥ 2 and < 5**	Mother's BMI	-3.110	0.064	0.006	0.115	8.298	0.006

	Mother's height		0.089	0.001			
**≥ 5**		-15.009			0.171	8.434	0.001
	Income		0.349	0.058			

**Table 5:** Multivariate analysis of independent variables in relation to the response variable (height-for-age), using the socioeconomic index income, of Xavante children < 10 years of age, according to age, Pimentel Barbosa village, Brazil, 2006.

Age in years	Independent variables	Linear coefficient	Angular coefficient	p-value	Adjusted R^2^	F-test	p-value (F-test)
	Age		-0.078	0.000			
**< 2**		-1.770			0.345	12.067	0.000
	Proportion of adults		4.845	0.030			

**≥ 2 e < 5**	Mother's BMI	-3.110	0.064	0.006	0.115	8.298	0.006

	Mother's height		0.095	0.000			
**≥ 5**		-16.038			0.196	9.761	0.000
	Wealth		0.439	0.020			

Maternal nutritional status, evaluated using BMI, was positively associated with the linear growth of children ≥ 2 and < 5 years of age, explaining 11.5% of the variability of the z-scores for the H/A index in both models, controlling for socioeconomic status.

As shown in Table 5, wealth and maternal height explained 19.6% of the variation observed in the dependent variable (H/A) for children ≥ 5 years old. Thus, children residing in households pertaining to the higher wealth stratum had, on average, H/A z-scores 0.439 higher than those in the lower stratum. The relationship was also positive with respect to maternal height. In this case, for every 1.0 cm increase in maternal height, an increase of 0.095 was observed in the child's z-scores for H/A (Table 5). Also for children ≥ 5 years old, income was included in the other model, despite the p-value being greater than 0.05 (p = 0.058), because it explained 17.1% of the variation observed in the response variable (Table 4).

Of the 232 children for whom W/A was calculated, 173 (74.6%) had sufficient data for regression analysis. In this case, no significant associations were encountered between any of the independent variables and W/A for any age group (data not shown).

## Discussion

The prevalences of W/A and H/A deficits in Xavante children under ten years of age from Pimentel Barbosa village were 5.6% and 14.7%, respectively, according to the NCHS. Regarding children under five years old, the prevalences of W/A and H/A deficits were 9.7% and 18.9%, respectively, according to the NCHS, and were 4.5% and 29.9%, respectively, according to the WHO. Although these values are notably elevated, studies conducted with other ethnic groups in the Amazon region of Brazil have reported substantially higher prevalences in both children under 10 years and under 5 years of age, particularly for low H/A, which often affects over 25% of evaluated children [4-6,25-27]. Previous nutritional surveys conducted among children under ten years of age in other Xavante communities showed similar weight and height deficits to those reported here [15,16].

The results of the present study reveal a substantially unfavorable nutritional scenario for the Xavante children of Pimentel Barbosa, especially those less than 5 years of age. Low H/A affects almost 30% of children within this age range (according to the WHO). This rate is greater than that documented by the First National Survey of Indigenous People's Health and Nutrition, which identified a height deficit of 26.0% for indigenous children in this age range nationally and 27.8% for indigenous children living in the Central-West region, where the Pimentel Barbosa Indigenous Reserve is located [7]. The greater deficits observed in the present study for children under 5 years as compared to those for children under 10 years of age is expected, given that the younger group is more vulnerable to nutritional disorders and associated morbidities, such as diarrhea and pneumonia [24,28,29].

As for W/A deficit, the prevalence observed in the present study for children under 5 years old (4.5%) is similar to that reported for indigenous populations in the Central-West region of Brazil (5.0% for children < 5 years of age) by the recent First National Survey of Indigenous People's Health and Nutrition [7]. In the present study, no instances of W/H deficit were observed. Considered together, the results obtained for the anthropometric indices constructed on the basis of weight, whether in reference to age or height, are similar to those reported in the literature for indigenous children from other ethnic groups in the Amazon and Central-West regions of Brazil, which show low or moderate prevalences of W/A deficit and practically no W/H deficits [26,27,30]. Even where high prevalences of weight deficit have been observed, as in the case of the Wari' from Rondônia [5,6], there was also a tendency to maintain body proportionality, which explains the rarity of cases of low W/H in children. The patterns observed in the present study are also consistent with those reported since the mid-1980s for non-indigenous children throughout Latin America, which led to genetic factors being ruled out as determinants of stunting in favor of the interpretation that socioeconomic factors, mother's education, housing, and sanitation play more prominent roles in explaining chronic undernutrition (stunting) among small children (see Victora [31] for a critical review on the matter).

It is worth noting that the frequencies of height and weight deficits reported in this study are substantially greater than those observed for Brazilian children nationally. According to a recent national survey of household economies (Pesquisa de Orçamentos Familiares), conducted in 2008-2009 [3], 6% of Brazilian children under 5 years of age show H/A deficits, according to the WHO reference. Recent analyses demonstrate a clear pattern of reductions in nutritional deficits in Brazilian children in recent decades, which may be explained by improved sanitation, increased health service coverage, greater access to education, and more equitable income distribution, among other factors [2]. An anthropometric survey carried out in Pimentel Barbosa village in May of 1994 indicated a prevalence of low H/A in children < 4 years of age of 22.0%, using the NCHS reference [14], which is similar to the present study (19.6%, as calculated from Table 2). Thus, there has not been a significant decrease in the prevalence rates of low H/A in small children from Pimentel Barbosa over a 12 year period. The nutritional scenario of the Xavante children described in the present study reveals a significant disparity relative to the national Brazilian population, which is consistent with the precarious sanitary conditions observed in Xavante communities and the low effectiveness and efficiency of health services provided to them, resulting in high levels of morbi-mortality by preventable infectious diseases [9,14,17,19].

The results of both regression models performed in this study demonstrate a positive relationship between the proportion of adults in a household and H/A in children younger than two years of age. Theoretically, a higher proportion of adults in the household could also result in less food availability for children. However, in the Xavante case it is associated with improved child growth. Overall, this result highlights the importance of the mediating relationship performed by the close family environment, especially in terms of food and hygiene. In this respect, the composition of Xavante households is an important dimension to be considered in the analysis of nutritional determinants because these are the primary social and economic units through which food is obtained, water is retrieved, and children are cared for in a more general sense. Therefore, the presence of a larger or smaller number of adults in a household may influence the nutritional status and overall health of its inhabitants, especially children. However, these results may be understood more specifically in terms of the relationship between household composition and childcare in Xavante society, as we describe below.

Studies conducted with non-Indian Brazilian children in different regions of the country point to the mediating role of family, emphasizing not just the mother's role in caring for children, but also the role of the entire residential group in contributing to their support and protection [32-34]. However, it is important to note that the Brazilian households predominantly addressed in these studies tend to be composed of nuclear families with small numbers of aggregates (uncles, aunts, grandparents, etc.). In contrast to this profile, Xavante domestic groups commonly consist of large extended families, which include matrilineal relatives from multiple generations (grandmothers, mothers, and daughters) and their respective spouses, with or without children. It is not uncommon for three or more nuclear families to reside in the same household. In this sociocultural setting, newlyweds often reside in the bride's parents' house after marriage (uxorilocal residence). A preference for sororal polygamy is also observed, whereby multiple sisters of different ages marry the same man [35]. Additionally, it is common for men and women to marry their siblings-in-law, a pattern that often results in the simultaneous coresidence of adult brothers and sisters. These patterns favor the coresidence of women and men of different ages, who share the responsibilities of childcare and resource provisioning. Of particular relevance to the care of young children, breast feeding may be performed by aunts or grandmothers, often members of the same household, when the mother is unavailable [36].

Within the age group ≥ 2 and < 5 years, only maternal nutritional status, expressed as BMI, showed a positive association with child's linear growth. In the group ≥ 5 and < 10 years of age, maternal height, in combination with income and wealth separately, explained 17.1% and 19.6% of the variability in children's linear growth, respectively. Such a direct correlation between the nutritional status of mothers and linear growth in children is most likely explained by similar environmental and socioeconomic conditions [28,32,33,37].

Maternal height and socioeconomic status are variables that often appear in the epidemiological literature as explaining the nutritional status of children of different ages [32,38-41]. The lack of correlation in the present study between the linear growth of children ≥ 2 and < 5 years of age and indicators of household composition or socioeconomic status is noteworthy because such associations were observed for children in other age ranges. Specifically, associations were encountered between linear growth and the proportion of adults in the household in children < 2 years of age and household wealth in children ≥ 5 and < 10 years of age. This intermediate pattern may be evidence of a gradual transition from greater dietary dependence on female adult caretakers during the first years of a child's life to relative independence in later childhood, characterized by more direct dependence on the domestic dietary economy.

In the regression analyses, the socioeconomic index wealth was positively associated with H/A in children ≥ 5 and < 10 years of age. However, no associations were observed between wealth or income and W/A for any age group. These results should be interpreted not only in terms of the specific socioeconomic context of the Xavante people but also in light of recent studies on indigenous Amazonian populations that indicate an absence of clear and consistent relationships between socioeconomic status and child health. Some studies demonstrate a similar association to that observed in the present study between indicators of participation in the market economy and the H/A of indigenous children [42,43]. However, these results are not always consistent for all anthropometric indicators of children's nutritional status or between the different indigenous populations studied. For instance, in a study of Kaingang children from Southern Brazil, Kühl et al. [44] found a significant relationship between socioeconomic status and W/A, but not for H/A, which is a different pattern of association than that encountered in the present study. Furthermore, as highlighted by Godoy et al. [45], different socioeconomic indices do not always show the same associations with anthropometric indicators of nutritional status. For example, studies of the nutritional status of Tsimane' children from Bolivia showed divergent patterns when different measures of market participation were used [42-44,46]. Similarly, the two indices of socioeconomic status utilized in the present study did not follow a single pattern.

These heterogeneous results are likely attributable to a variety of factors. First, different health indicators may capture distinct aspects of child nutritional status. Deficits in H/A and W/A, in particular, reflect vastly different nutritional processes - chronic and acute undernutrition, respectively. Second, different measures of socioeconomic status may capture distinct aspects of differentiation processes. In this regard, Godoy and Cardenas [47] highlighted an apparent contrast in the literature between processes of market participation, arguing that the involvement of indigenous peoples in wage labor and agricultural production may produce opposite health effects. In the Xavante case, although both indices of socioeconomic status (wealth and income) are related to paid employment, they do not necessarily behave the same way in the regression models used to analyze nutritional status. Considering that wealth, but not income, was shown to be associated with children's nutritional status, the first index possibly captures with more sensitivity differences in food practices between Xavante households. A possible explanation for this contrast may be that the wealth index measures the long-term global consumption pattern of a household (including food) more directly than the income index. In other words, wealth therefore represents a cumulative process, just as linear growth is a measure of long-term nutritional status.

A prominent aspect of the Xavante domestic economy is a strong tendency for reciprocal sharing of resources between households. As a result of this pattern, high or low monetary income is not necessarily singularly related to the acquisition of industrial goods. As previously discussed for the same Xavante community [18], there are strong indicators of emergent internal socioeconomic differentiation. However, the manner in which each household is inserted in the market economy is also related to non-monetary forms of food acquisition and consumption, including reciprocity.

## Conclusions

This study demonstrates that undernutrition, in particular linear growth deficit, is a notable health issue for Xavante children. Low height-for-age was associated with household composition in the youngest age group analyzed (< 2 years of age) and with household wealth in the oldest age group (≥ 5 and < 10). These data reaffirm the relevance of undernutrition, particularly as indicated by linear growth deficit, in characterizing the nutritional profile of indigenous children in Brazil. The frequencies of undernutrition observed in Xavante children from Pimentel Barbosa village, which were substantially higher than the averages reported for the Brazilian national population, call attention to the persistent health disparities that exist between indigenous and non-indigenous people in the country. In this context, the continual monitoring of physical growth should be considered a strategic tool for evaluating the health conditions of indigenous children, as well as for assessing the possible determinants of child nutritional status. Its inclusion in the routine of local health services would contribute to the advancement of nutritional interventions in this segment of the population known to be particularly vulnerable to health effects of food insecurity and poor sanitation.

## Competing interests

The authors declare that they have no competing interests.

## Authors' contributions

AAF, JRW, CEAC, and SAG participated in the conception of the study, collection of data in the field, data analysis, and drafting of the manuscript. RVS and SAG contributed to the study design, interpretation of the data, and writing of the paper. All authors participated in the revision of the manuscript and approved the version submitted for publication.

## List of Abbreviations

ANOVA: Analysis of variance; H/A: Height-for-age; NCHS: National Center for Health Statistics; SPSS: Statistical Package for the Social Sciences; VIF: Variance inflation factor; W/A: Weight-for-age; W/H: Weight-for-height; WHO: World Health Organization.
